# ADMISSIONS AND DISCHARGE PRACTICES IN ASSISTED LIVING: THE ROLE OF REGULATORY AND ORGANIZATIONAL CHARACTERISTICS

**DOI:** 10.1093/geroni/igad104.0213

**Published:** 2023-12-21

**Authors:** Cassandra Hua, Lindsey Smith, Sheryl Zimmerman, Gauri Gadkari, Philip Slolane, Paula Carder, Christopher Wretman, Kali Thomas

**Affiliations:** University of Massachusetts Lowell, Lowell, Massachusetts, United States; Oregon Health & Science University, Portland, Oregon, United States; University of North Carolina at Chapel Hill, Chapel Hill, North Carolina, United States; Brown University School of Public Health, Providence, Rhode Island, United States; University of North Carolina School of Medicine, Chapel Hill, North Carolina, United States; Portland State University Institute on Aging, Portland, Oregon, United States; Wretman LLC, Hillsborough, North Carolina, United States; Johns Hopkins University, Baltimore, Maryland, United States

## Abstract

This study examined how state regulations and AL organizational characteristics relate to admissions and discharge practices. Using data from a representative sample of 250 AL communities in 7 states and a database of AL regulations, we employed bivariate analysis and multilevel linear probability models to examine regulatory and organizational correlates of AL community admissions and discharge practices for three activities of daily living (ADLs [bathing, getting out of bed, and eating]). There was a pattern between admissions regulation and admissions practices in AL; for example, 38% of communities with license types that had the most flexible admissions regulations allowed residents with feeding limitations to be admitted; conversely, 18% with the least flexible regulations allowed them to be admitted. However, the relationships were not statistically significant in bivariate or multivariate analysis. A higher percent of residents with Medicaid as a primary payer source was associated with a decrease in the probability of admitting (𝛽 = -0.01, p<.001, 95% confidence intervals [CI]: -0.01, 0.00) and discharging (𝛽 = 0.01, p=.013, 95% CI: 0.00, 0.01) residents with bathing limitations. Being operated in association with a continuing care retirement community or nursing home was associated with a higher probability of discharging residents who needed assistance with bathing (𝛽 = 0.14, p=.013, 95% CI: 0.03, 0.26) and more so with feeding (𝛽 = 0.26, p <.001, 95% CI: 0.15, 0.36). Findings suggest a need to consider whether models of AL and AL practices could better align with the residents’ needs.

